# Zincbindpredict—Prediction of Zinc Binding Sites in Proteins

**DOI:** 10.3390/molecules26040966

**Published:** 2021-02-12

**Authors:** Sam M. Ireland, Andrew C. R. Martin

**Affiliations:** Division of Biosciences, Institute of Structural and Molecular Biology, University College London, Darwin Building, Gower Street, London WC1E 6BT, UK; sam.ireland.09@ucl.ac.uk

**Keywords:** zinc, metal binding, proteins, prediction, machine learning

## Abstract

**Background:** Zinc binding proteins make up a significant proportion of the proteomes of most organisms and, within those proteins, zinc performs rôles in catalysis and structure stabilisation. Identifying the ability to bind zinc in a novel protein can offer insights into its functions and the mechanism by which it carries out those functions. Computational means of doing so are faster than spectroscopic means, allowing for searching at much greater speeds and scales, and thereby guiding complimentary experimental approaches. Typically, computational models of zinc binding predict zinc binding for individual residues rather than as a single binding site, and typically do not distinguish between different classes of binding site—missing crucial properties indicative of zinc binding. **Methods:** Previously, we created ZincBindDB, a continuously updated database of known zinc binding sites, categorised by family (the set of liganding residues). Here, we use this dataset to create ZincBindPredict, a set of machine learning methods to predict the most common zinc binding site families for both structure and sequence. **Results:** The models all achieve an MCC ≥ 0.88, recall ≥ 0.93 and precision ≥ 0.91 for the structural models (mean MCC = 0.97), while the sequence models have MCC ≥ 0.64, recall ≥ 0.80 and precision ≥ 0.83 (mean MCC = 0.87), with the models for binding sites containing four liganding residues performing much better than this. **Conclusions:** The predictors outperform competing zinc binding site predictors and are available online via a web interface and a GraphQL API.

## 1. Introduction

Many proteins require a cofactor to function correctly, and present a region of their surface which has an affinity for that cofactor. Of the metallic cofactors, zinc is one of the most common. Approximately 10% of proteins require zinc to function [1] and so have at least one zinc binding site, making it the second-most prevalent metal in biological systems, after iron. In proteins, it typically performs either a rôle in catalysis (despite, or more likely because of, its lack of variable redox states), or in stabilising a region of the protein [2].

While there are many proteins which are known to bind zinc because the full three-dimensional structure of the protein has been solved in the presence of zinc, leading to the identification of a zinc binding site, it would be useful to be able to determine whether a protein binds zinc without needing to do this. There are experimental means of doing so, but computational approaches offer a more convenient means of performing initial searches at greater scale and speed. These would take either the protein’s sequence, or a structure of some kind (either a hypothetical model, an experimental structure generated in the absence of zinc, or an experimental structure solved at low resolution where a zinc cannot be identified, perhaps because of the presence of heavy metals used for isomorphous replacement) and try to predict whether the protein binds zinc, and, if so, where.

There have been numerous studies in this area in the past. Early attempts at predicting zinc binding from sequence were largely done manually, such as by identifying the ‘C…C…H…H’ (cys-cys-his-his) motif as being a characteristic indicator of zinc binding [3,4], or by identifying approximate spacing patterns typical of catalytic binding sites—the so-called ‘short and long spacers’ [5]. As the number of available sequences grew and this manual approach became infeasible, sequence alignment with known zinc binding proteins became a useful tool for discovering new zinc binding sites [6,7]. Resources such as PROSITE [8] provide a refinement of manual motif searching by providing motifs for zinc binding in a number of homologous families. At the time of writing, there are 70 motifs for zinc fingers, one for zinc-containing alcohol dehydrogenases, two for copper/zinc superoxide dismutase signature, two for zinc carboxypeptidases and one for the zinc import ATP-binding protein znuC family.

By the early 2000s, machine learning became the typical approach for identifying possible metal binding sites—a collection of algorithms which are trained on a dataset of known zinc binding sites in order to identify for themselves what the characteristic properties of zinc binding are, rather than having a human manually identify what those properties might be. Typical algorithms used in the past include Support Vector Machines (SVMs) [9,10,11] and Random Forests [12,13]. In recent years, deep learning, which relies on multi-layer neural networks to represent the inputs at multiple layers of abstraction, has been used more widely [14,15].

Predicting zinc binding from structure has proceeded in a similar fashion, although the nature of structural data means that it has taken longer for there to be enough data to justify the use of machine learning techniques. Early efforts relied on human-observed characteristics of zinc binding sites, such as the ‘hydrophobicity contrast function’, which used the fact that metal binding sites tend to be composed of an inner shell of hydrophilic atoms such as nitrogen and sulphur, which was, in turn, surrounded by a stabilising shell of hydrophobic atoms [16,17]. As the number of available structures grew, geometric patterns were also observed—both by humans and by machine learning algorithms [17,18,19,20]. As with the sequence prediction models, the complexity of the algorithms, and of the zinc binding site features, has grown with the increase in available training data.

One recurring feature, particularly in the sequence-based predictive models, is the focus on zinc binding *residues* rather than zinc binding *sites*. In most cases, the entity examined by the predictive model is the individual residue, often with a surrounding linear sequence ‘window’ of residues. The model then assigns a probability as to whether that residue is a zinc binding residue. As outlined above, this approach has had a measure of success, but it is a somewhat artificial concept. There is, after all, no such thing as a zinc-binding residue in isolation. The individual residues of a high-affinity zinc binding site of the kind considered here are only zinc-binding when the other residues are present, and conversely many non-zinc-binding residues could bind zinc if other residues were present in the correct locations. It is particular *combinations* of residues, not individual residues, which are zinc binding—an important fact not usually considered in research of this kind.

Another commonality is the treatment of zinc binding sites as a single category, and the presumption of properties that are common to them all regardless of the residues of which they are comprised. This may well be sufficient—particularly as there are essentially only four residues that make up the vast majority of zinc binding sites—but it is possible that properties used for prediction have much tighter distributions within particular sub-categories of zinc binding sites.

Previously, we created ZincBindDB [21], a database of zinc binding sites. This resource continuously collates all zinc atoms found in the Protein Data Bank [22], identifies their binding sites (where appropriate), and stores them in a centralised database along with useful properties such as their protein sequence and how different sites cluster together. Sites are classified into ‘families’, not based on homology, but based on the residue composition of the site—the C4 family contains binding sites with four cysteines, H3 those with three histidines, and so on. These data are available over the web via a web ‘application programming interface’ (API), and using a web interface which provides three dimensional graphical representations of all the binding sites. As of July 2020, there were 35,811 zinc binding sites in ZincBind, originating from 16,635 PDB structures.

We have now used this single, definitive dataset of zinc binding sites to train predictive models of zinc binding. Here, we present models which are trained to detect entire zinc binding sites, rather than just zinc binding residues, and each predictive model is trained to detect a particular family of zinc binding sites. There are distinct models for sequence and for structure, and predictions can be made via the ZincBind website, or via the ZincBindPredict GraphQL API.

## 2. Results and Discussion

### 2.1. Deployment

**Figure 1:**
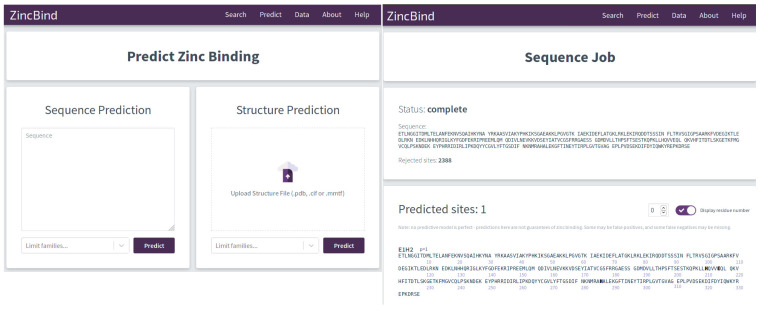
The graphical interface for the predictors is shown on the left. The user can enter a protein sequence, or upload a structure file. In both cases, the user has the option of limiting the zinc binding families for which the predictor will search, which can save a considerable amount of time. Results of the prediction are shown on the right with the residues predicted to form a binding site shown in bold. This interface consumes the ZincBindPredict GraphQL application programming interface (API), which is also publicly available.

The trained predictive models are available via a simple web interface at https://zincbind.bioinf.org.uk/predict/ (see Figure 1). This takes a sequence or an uploaded PDB file and scans it against each of the models, reporting whether any of them suggest a zinc binding site. Alternatively, the ZincBindPredict GraphQL API may be accessed directly. A GraphQL request can be sent with either a protein sequence or protein structure, and a job ID will be returned. This can then be polled for results as the protein or sequence is searched using each model in turn, with the identified binding sites returned as a list with the associated probability.

### 2.2. Training

For all twenty datasets (sequence and structure sets each with 10 different combinations of liganding residues), the ratio of positive samples (actual binding sites) to negative samples (combinations of residues matching a zinc-binding site family, but which are known not to bind zinc) was approximately 1:1. The dataset sizes ranged from 804 to 15,332 samples for the sequence datasets, and from 407 to 3232 samples from the structure datasets.

### 2.3. Models

Model effectiveness was measured using recall, precision, F1 score, and Matthews Correlation Coefficient (MCC) for all twenty models (10 structural and 10 sequence).

For the structural models, the lowest MCC score was 0.88 (for the E1H1 model). This, and the D1H1 model (MCC = 0.91), relies on the geometry between just two residues, which makes creating a distinct separation between the two classes somewhat more difficult—though their performance is still very close behind that of the three- and four-residue family models. The structure models had an average MCC of 0.97 (see Table 1).

The sequence models also had high scores, though were more variable. The four residue sites in particular had highly conserved patterns of residue spacing and flanking hydrophobicity despite being from several homologous families. The average MCC for the sequence models was 0.87, with the lowest MCC being 0.61 for the E1H1 model and 0.74 for the D1H1 model—again the two two-residue models were somewhat behind the MCC of 0.84 for the C3 model (see Table 2).

**Figure 2:**
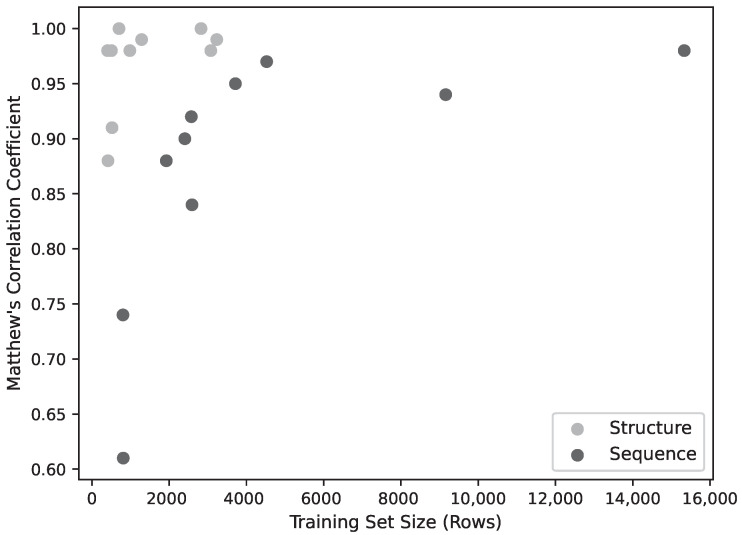
Model Performance (MCC) as a function of training set size. Below ∼4000 training patterns, performance declines sharply, though above this threshold there ceases to be a strong correlation between performance and training set size.

**Figure 3:**
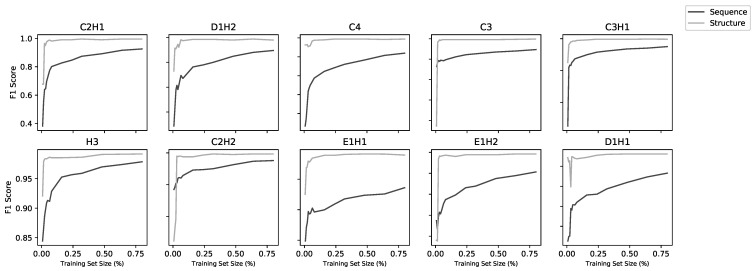
Learning curves for all 20 models (10 structural and 10 sequence). Each model was trained on increasing subsets of the overall training set using five-fold cross-validation. Sequence models improved with increasing dataset size whereas, above a low threshold, structure models did not improve with more data.

While the training is affected by dataset size, this does not appear to be a significant limiting factor for most of the models. Figure 2 shows the model performance (as MCC) for the sequence and structure models. The performance of the sequence models falls off as the training set size falls below ∼4000, while the performance of the structural models falls off below around 1000 data points. The lowest three performing structural models were also the lowest three in dataset size (C3, E1H1, D1H1), but two of these have only two residues so, as discussed above, the performance might not be expected to be very good. Learning curves (Figure 3) using fractions of the datasets show a correlation with dataset size for the sequence models, but above around 1000 sequences, the structure models do not improve with larger datasets.

The level of abstraction used to describe both sequences and structures (see Section 3 Methods) made it unlikely that any homology between data in the training and testing sets would artificially improve the performance. The features are largely calculated from residues around the binding residues, rather than the sequence in which they occur. Nonetheless, we confirmed that this was true.

Different sequence identity thresholds were used for clustering with CD-HIT and, where possible, a dataset of the same size was selected at random from each set of resulting clusters. No significant effect on performance was seen. When clustering at 40% sequence identity, there was slightly lower performance (see Supplementary File clustering.txt), but clustering at this level did result in much smaller datasets. As indicated previously, this is a major determinant of the performance of the sequence models.

**Figure 4:**
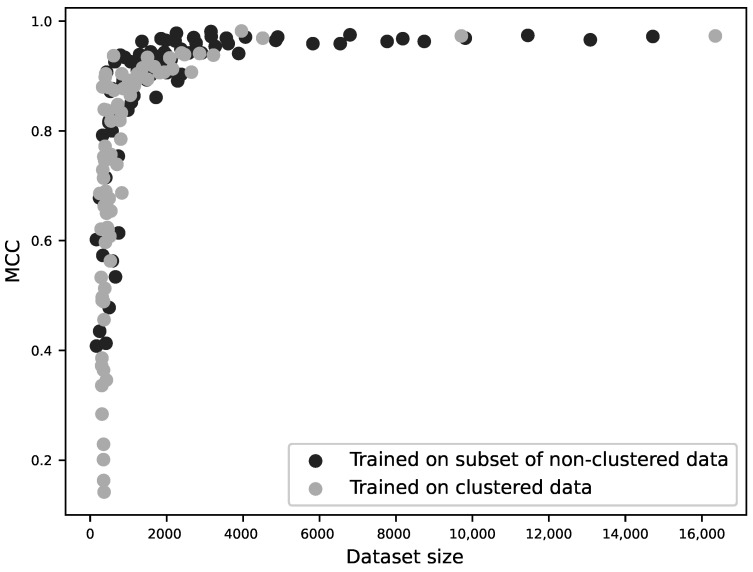
MCC as a function of dataset size for 160 different sequence-based models. For each of the ten zinc-binding site families, 9 classifiers were trained using 20–100% of the original, unclustered data (10 × 9 models); additional classifiers were trained using sequences clustered at 40–100% sequence identity (10 × 7 models). The performance (MCC) is plotted against the size of the training dataset. The two modes of dataset reduction are shown by different shades and it can be seen that the curves are not significantly different. This suggests that homology between training and test sets does not influence a model’s performance; rather, performance is a function of training dataset size.

In order to identify whether this lowered performance was because the models performed worse without the possibility of homologous sequences between the training and test sets, or whether it was a result of the smaller training set, for each zinc-binding site family, a classifier was trained on 20%, 30%, 40%, 50%, 60%, 70%, 80%, 90% and 100% of the original, unclustered data, and additional classifiers were trained on data with sequences clustered at 40%, 50%, 60%, 70%, 80%, 90% sequence identity and with no clustering. The performance of the models was then plotted against the resulting dataset sizes as shown in Figure 4. This demonstrates that it is dataset size that determines model performance, regardless of any similarity of the sequences between the training and testing datasets.

For reference, the performance of the sequence models was compared with using BLAST for predicting zinc-binding sites. For each zinc-binding site family, a BLAST database was created using 80% of the available zinc-binding sequences, and BLAST’s ability to identify zinc binding sites from the remaining 20% was compared against an equivalently sized negative set. The results are shown in Table 3. With the exception of C2H2, using BLAST to find zinc binding based on homology performs much worse than the models presented here. Even in the case of C2H2, which seems to have much more similar sequences in its dataset, the ZincBindPredict model still narrowly outperforms BLAST.

The performance scores of our predictors also compare favourably with recent comparable predictive models based on structure and sequence—most notably the ‘SVM and Sample-Weighted Probabilistic Neural Network’ (MCC = 0.80) [11], the ‘meta-zinc predictor’ (MCC = 0.79) [23] and ZincExplorer (MCC = 0.78) [24].

However, the models presented here are not intended to be general-purpose zinc binding predictors that detect common properties of all zinc binding sites—they are zinc-binding site family-specific predictors based on the principle that common, specific types of zinc binding site have more identifiable, consistent properties than do zinc binding sites in general. As a result, they will not readily detect binding sites of uncommon zinc-binding families. This abstract predictiveness has been deliberately discarded to create highly effective models for specific, common families of zinc binding sites. It is also noteworthy that the binding site itself is a useful unit of prediction using this methodology—even for sequences—rather than individual binding residues. The models are therefore identifying something biologically real (a zinc binding site) rather than something which does not actually exist in isolation (a single zinc binding residue), but which is a useful heuristic in some circumstances.

A demonstration of this can be seen by applying the sequence models to bacterial genomes to measure the proportion of typical genomes that the models predict to be zinc binding, as shown for a range of bacterial genomes in Table 4. For most genomes, fewer than 10% of proteins are flagged as zinc binding, with the average for the genomes examined being 8.46%. Given that the zinc-binding families for which predictors have been generated represent 67.0% of binding sites in ZincBindDB (the others being unusual sites), this would imply a ‘true’ predicted proportion of 12.6%, which is a little higher than the widely cited figure of 10%.

## 3. Materials and Methods

### 3.1. Dataset Creation

The datasets used to train the predictive models were derived from ZincBindDB.

For the sequence models, for each family of zinc binding sites, all examples were downloaded with the associated sequences, and those with more than one sequence (those sites split across multiple chains) were discarded. The resulting sequences were turned into feature vectors which contained the number of residues between each pair of binding residues, the average hydrophobicity of residues either side of the binding residues, using the features described in Table 5. This created a dataset of positive samples. For the negative samples, for each zinc-binding site family, a sequence was chosen at random from the set of all unique sequences in UniProtKB and a combination of residues within that sequence matching the zinc-binding site family (e.g., C2H2), but not a known binding site, was selected—this was done repeatedly until a list of negative samples was built up equal in size to the positive dataset. The two datasets were combined into a single dataset for each zinc-binding site family.

For the structural data, for each zinc-binding site family, all relevant zinc binding sites belonging to a PDB structure with resolution better than 2 Ångströms were downloaded, and grouped by the PDB entry to which they belonged. For each PDB entry, the structure was downloaded and parsed using the Python library atomium [27], assembled into the correct biological assembly, and then each binding site was turned into a feature vector using the features described in Table 5. Since the distances used are all the pairwise combinations of the atoms involved, the number of distances depends on the number of liganding residues: H3 sites will have three inter Cα distances, C4 sites will have six, and so on. The ‘hydrophobicity contrast function’ is calculated at the centre of the Cβ atoms with a radius of 4 Ångströms as described in the original paper by Yamashita et al. [16]. This algorithm is a measure of how much outer atoms in a sphere are more hydrophobic than inner atoms, with higher values previously shown to be associated with centres of metal binding [16,17].

For example, given a C2H2 site, in a sequence model, there would be three inter-residue gaps for which the number of residues per gap would be used together with the mean hydrophobicity and charge of the 4 interacting residues (i.e., a window of 1), the 4 interacting residues plus one sequence neighbour on each side (window of 3) and the 4 interacting residues plus two sequence neighbours on each side (window of 5). For the structural model, there would be 6 inter-Cα and 6 inter-Cβ distances, from which the mean, maximum, minimum and standard deviation would be calculated as well as the hydrophobicity contrast function.

To generate the negative samples, for each positive sample, a random arrangement of residues matching the zinc-binding site family in question was obtained from a randomly chosen, non-zinc-binding PDB structure, and a feature vector created from that non-binding combination. In this case, only residue combinations that could feasibly form a binding site (those where there are no inter-Cα distances greater than 30 Ångströms) were used.

While the abstraction of sequence and structure suggests that homology is unlikely to influence the results (i.e., homologues between training and testing sets are unlikely to over-rate the performance), this was tested using datasets with similar sequences removed. CD-HIT [28] was used with sequence identity cutoffs ranging from 100% down to 40% (the lowest identity threshold for the standard version of CD-HIT).

### 3.2. Predictive Model Training

The Random Forest algorithm [29] was used to train the predictive model for each of the 20 datasets (a dataset of sequence features and a dataset of structural features for each of the ten zinc binding site families), which provided superior results to K-Nearest Neighbours, and vastly superior results to Support Vector Machines even when the dataset was balanced. Random Forests apply the bagging concept (where multiple models are trained on random sub-samples of the data to avoid over-fitting to the training data) to decision trees (classification algorithms which divide the input space into the categories based on sequential binary splits).

The hyper-parameters for each model were selected separately using 5-fold cross validation of the training set. The hyper-parameters explored were the impurity measure (gini *vs.* entropy—the algorithm used to split individual trees at each node), the maximum depth that the component trees could have (4, 6, 8 or no maximum), the number of trees in the forest (10, 100 or 1000), and the means of determining the best number of features at each split (either the square root of the number of features, or the log${}_{2}$ of the number of features). Once optimal hyper-parameters were identified (determined by which combination produced the best F1 score in the cross-validation), the models were trained with those hyper-parameters using the entire training dataset.

For the trained model, the metrics recall (how effective at finding true binding sites the models are), precision (how effective at ignoring non-binding sites they are), the F1 score (the harmonic mean of recall and precision) and Matthews Correlation Coefficient (another summary of the true positives, true negatives, false positives and false negatives generally considered the best overall metric [30]) were calculated using the separate test datasets (the test–train split being 20:80). The accuracy metric was not used as it is not relevant for unbalanced datasets. Training was performed using the Python scikit-learn library [31].

For performance comparison, homology searching was performed using the NCBI BLAST program [32] using an expectation value threshold of 0.1.

## 4. Conclusions

Zinc binding sites can be divided into distinct families based on the residues of which they are comprised. These zinc-binding site families follow a power law distribution, with a small number of families being highly represented. By training models for individual zinc-binding site families, rather than for zinc binding sites in general, very high recall and precision levels can be achieved. It is worth noting that a zinc-binding site family is a completely different concept from a homologous family as it is the result of convergent evolution potentially spanning many different homologous families. The high performance suggests that, for both sequence and structure, zinc binding properties are more tightly distributed within zinc binding site families than for zinc binding sites generally. The resulting predictor outperforms other general zinc binding predictors. 

## Figures and Tables

**Table 1:** Results for structure models, sorted by Matthews Correlation Coefficient (MCC). The two-residue families’ performance was lower than the others as there are essentially just the measurements between two centres to perform the classification, but still scored relatively highly. Four-residue sites in particular were found to have very high performance.

Family	Dataset Size	Recall	Precision	F1	MCC
C2H2	702	1.00	1.00	1.00	1.00
C4	2825	1.00	1.00	1.00	1.00
C3H1	3232	1.00	0.99	1.00	0.99
E1H2	1287	1.00	0.99	1.00	0.99
C2H1	506	1.00	0.98	0.99	0.98
H3	3078	1.00	0.98	0.99	0.98
D1H2	982	1.00	0.98	0.99	0.98
C3	407	1.00	0.98	0.99	0.98
D1H1	522	1.00	0.91	0.95	0.91
E1H1	416	0.93	0.95	0.94	0.88
Mean		0.99	0.98	0.99	0.97

**Table 2:** Results for sequence models, sorted by Matthews Correlation Coefficient (MCC).

Family	Dataset Size	Recall	Precision	F1	MCC
C4	15,332	1.00	0.98	0.99	0.98
H3	4524	0.98	0.99	0.98	0.97
C2H2	3715	0.97	0.99	0.98	0.95
C3H1	9158	0.98	0.96	0.97	0.94
E1H2	2574	0.95	0.97	0.96	0.92
D1H2	2406	0.94	0.95	0.94	0.90
C2H1	1926	0.93	0.95	0.94	0.88
C3	2591	0.95	0.89	0.92	0.84
D1H1	804	0.80	0.93	0.86	0.74
E1H1	812	0.81	0.83	0.82	0.61
Mean		0.93	0.94	0.94	0.87

**Table 3:** Predictive ability of BLAST to identify zinc binding sites in protein sequences using homology alone.

Family	Dataset Size	Recall	Precision	F1	MCC
C2H2	3960	0.99	0.95	0.97	0.94
C3H1	9710	0.29	0.87	0.44	0.33
C2H1	2154	0.24	0.88	0.37	0.30
D1H1	818	0.05	0.80	0.09	0.11
C3	2868	0.13	0.61	0.21	0.07
E1H1	828	0.06	0.62	0.11	0.06
D1H2	2470	0.03	0.53	0.06	0.01
H3	5058	0.01	0.19	0.02	−0.10
E1H2	2648	0.02	0.33	0.04	−0.06
Mean		0.18	0.58	0.23	0.17

**Table 4:** Percentage of protein sequences encoded in the genome predicted to be zinc binding by ZincBindPredict for an assortment of bacterial genomes. Genomes were acquired from ensembl [25] in the form of translated polypeptide sequences, with a sequence labelled as zinc binding if any of the ten models finds at least one zinc binding site for that sequence/family combination. See Supplementary File genomes.zip for the full results.

Species	Percentage of Genome
	Predicted Zinc Binding
*Campylobacter jejuni*	6.4%
*Clostridioides difficile*	5.8%
*Enterococcus faecalis*	7.5%
*Listeria monocytogenes*	7.9%
*Mycobacterium tuberculosis*	11.3%
*Salmonella enterica*	11.1%
*Shigella flexneri*	10.1%
*Streptococcus pneumoniae*	7.6%

**Table 5:** Details of how features are calculated for residue combinations in structure and sequence models. Hydrophobicity of sequence residues is defined using Wimley and White’s scale [26], charge is the count of charged residues (aspartate, glutamate, arginine, histidine and lysine).

Model Type	Feature
**Sequence**	
	Inter-residue distance (one per gap)
	Average hydrophobicity around residues (window 1)
	Average hydrophobicity around residues (window 3)
	Average hydrophobicity around residues (window 5)
	Average number of charges around residues (window 1)
	Average number of charges around residues (window 3)
	Average number of charges around residues (window 5)
**Structure**	
	Mean Inter-Cα distance
	Maximum Inter-Cα distance
	Minimum Inter-Cα distance
	Inter-Cα distance standard deviation
	Mean Inter-Cβ distance
	Maximum Inter-Cβ distance
	Minimum Inter-Cβ distance
	Inter-Cβ distance standard deviation
	Hydrophobic contrast (radius 4 Å)

## Data Availability

The resource may be accessed at https://zincbind.bioinf.org.uk/predict/ and the source code at https://github.com/samirelanduk/ZincBindPredict.

## Supplementary Materials

The following are available online.

## Abbreviations

The following abbreviations are used in this manuscript:

API	Application Programming Interface
SVM	Support Vector Machine
PDB	Protein Data Bank
MCC	Matthews Correlation Coefficient
